# RNA-Seq analysis reveals transcript diversity and active genes after common cutworm (*Spodoptera litura* Fabricius) attack in resistant and susceptible wild soybean lines

**DOI:** 10.1186/s12864-019-5599-z

**Published:** 2019-03-22

**Authors:** Haiping Du, Xiao Li, Lihua Ning, Rui Qin, Qing Du, Qing Wang, Haina Song, Fang Huang, Hui Wang, Deyue Yu

**Affiliations:** 1 National Center for Soybean Improvement, National Key Laboratory of Crop Genetics and Germplasm Enhancement, Jiangsu Collaborative Innovation Center for Modern Crop Production, Nanjing Agricultural University, Nanjing, 210095 China; 2 Jiangsu Academy of Agricultural Sciences, Provincial Key Laboratory of Agrobiology, Institute of Germplasm Resources and Biotechnology, Nanjing, 210014 China; 3 College of Chemistry and Chemical Engineering, Key Laboratory of Ecological Restoration in Hilly Area, PingDingshan University, Pingdingshan, 467000 China

**Keywords:** Wild soybean (*Glycine soja* Sieb. & Zucc.), Resistance to insects, Common cutworm, RNA-Seq analysis, Phosphate transporter

## Abstract

**Background:**

Common cutworm (CCW) is highly responsible for destabilizing soybean productivity. Wild soybean is a resource used by breeders to discover elite defensive genes.

**Results:**

The transcriptomes of two wild accessions (W11 and W99) with different resistance to CCW were analyzed at early- and late-induction time points. After induction, the susceptible accession W11 differentially expressed 1268 and 508 genes at the early and late time points, respectively. Compared with W11, the resistant accession W99 differentially expressed 1270 genes at the early time point and many more genes (2308) at the late time point. In total, 3836 non-redundant genes were identified in both lines. Gene Ontology (GO) and Kyoto Encyclopedia of Genes and Genomes (KEGG) analyses revealed that the differentially expressed genes (DEGs) in W99 at the late time point were mostly associated with specific processes and pathways. Among the non-redundant genes, 146 genes were commonly up-regulated in the treatment condition compared with the control condition at the early- and late-induction time points in both accessions used in this experiment. Approximately 40% of the common DEGs were related to secondary metabolism, disease resistance, and signal transduction based on their putative function. Excluding the common DEGs, W99 expressed more unique DEGs than W11. Further analysis of the 3836 DEGs revealed that the induction of CCW not only up-regulated defense-related genes, including 37 jasmonic acid (JA)-related genes, 171 plant-pathogen-related genes, and 17 genes encoding protease inhibitors, but also down-regulated growth-related genes, including 35 photosynthesis-related genes, 48 nutrition metabolism genes, and 28 auxin metabolism genes. Therefore, representative defense-related and growth-related genes were chosen for binding site prediction via co-expression of transcription factors (TFs) and spatial expression pattern analyses. In total, 53 binding sites of 28 TFs were identified based on 3 defense-related genes and 3 growth-related genes. Phosphate transporter *PT1*, which is a representative growth-related gene, was transformed into soybean, and the transgenic soybean plants were susceptible to CCW.

**Conclusions:**

In summary, we described transcriptome reprograming after herbivore induction in wild soybean, identified the susceptibility of growth-related genes, and provided new resources for the breeding of herbivore-resistant cultivated soybeans.

**Electronic supplementary material:**

The online version of this article (10.1186/s12864-019-5599-z) contains supplementary material, which is available to authorized users.

## Background

Soybean [*Glycine max* (L.) Merr.] crops have substantial economic value and account for more than half of global oilseed production [1]. However, throughout their whole growth period, soybean plants are affected by many insects and diseases. The common cutworm (CCW), which belongs to the order Lepidopterans, is one of the major leaf-feeding insects in southern soybean regions in China [2]. As generalist herbivore insects, CCWs voraciously feed on many plants and can even cause 100% loss of soybean production if no control measures are taken [3]. Although the application of insecticides can reduce yield losses, the increased cost and residual insecticide in soybean seeds and plants can threaten the health of both humans and animals and can even cause environment destruction. Instead, the genetic improvement of soybean resistance to insects is an alternative for controlling insect damage.

Genetic improvement depends on the identification of insect resistance genes. Transcriptome sequencing is an effective method for unraveling the resource of genes and has been widely used in recent years. For example, using a transcriptome analysis, Hickman et al. identified 4 previously uncharacterized transcription factor (TF) genes that confer resistance to fungi and insects in *Arabidopsis*, and Wang et al. reported that *GmVSPβ* and *GmN:IFR* confer resistance to CCW in cultivated soybean [4, 5]. Furthermore, Huang et al. identified 1969 transcripts that were significantly differentially expressed after cotton bollworm attack in cotton [6]. Liu et al. identified 15,997 and 15,494 differentially expressed genes (DEGs) in the leaves and roots of soybean seedlings under salt stress, and Du et al. identified 655 MYC2-targeted jasmonic acid (JA)-response genes using RNA-Seq combined with chromatin immunoprecipitation coupled with high-throughput sequencing (ChIP-Seq) in tomato [7, 8]. However, the CCW-induced metabolic and regulatory gene networks in wild soybean are poorly understood.

Cultivated soybean was domesticated from wild soybean (*Glycine soja* Sieb. & Zucc.) in China 5000 years ago [9]. The transformation of the wild species into cultivars severely altered the morphology and phenotype of the plants to meet human demands under natural and human selections; these morphological and phenotypic traits included yield, apical dominance, seed size, growth habit, etc. However, the process narrowed the genetic diversity of soybean. A reduction in genetic diversity can result in “broad susceptibility” to newly emerging herbivores and pathogens [10, 11]. By analyzing the genetic diversity of 196 wild soybean lines and 200 landraces of cultivated soybean, Wen et al. reported that only 65.5% of the alleles of landraces were inherited from wild soybean [12]. By resequencing 62 wild soybean lines, 130 landraces, and 110 improved cultivars, Zhou et al. reported that wild soybean had the highest number of specific sequences, followed by landraces and improved cultivars. Approximately half of the annotated resistance-related sequences in wild soybean were lost in landrace and improved cultivar samples [13]. Wild soybean clearly houses a gene pool from which breeders could discover new elite genes. However, few studies have investigated the use of wild soybean for insect resistance [14].

In this study, two wild soybean accessions, including W11, which is susceptible to CCW, and W99, which is resistant to CCW, were chosen to test the progression of induced resistance levels after insect feeding. The early- and late-induction time points in the two accessions indicated by the expression patterns of two typical genes (*LOX7* and *VSPβ*) were chosen for the transcriptome analysis by RNA-Seq. By comparing the results of the analysis between the treated and control groups, we identified a severe DEG response to CCW attack and further analyzed the up-regulated defense-related and down-regulated growth-related genes. The functional analysis of a down-regulated phosphate transporter gene showed specific susceptibility in the overexpression transgenic soybean plants. We attempt to explain the mechanisms of soybean resistance to insects and provide resources for breeding soybean insect resistance in this study.

## Results

### Resistance to CCW and gene expression dynamics in W11 and W99

**Fig. 1:**
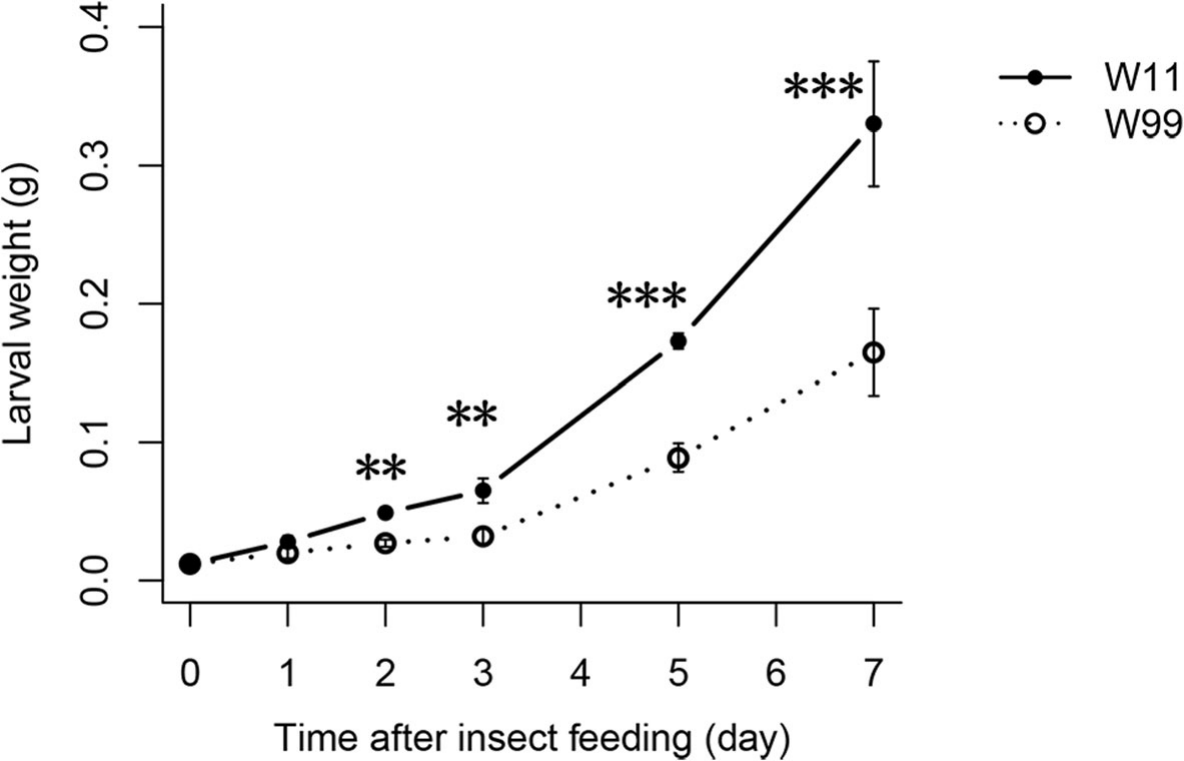
Dynamics of resistance to CCW based on larval weight for 7 consecutive d of feeding for the resistant line W99 and susceptible line W11. The larvae were weighed before feeding (0 d) and after feeding at 1, 2, 3, 5, and 7 d. The error bars represent the standard deviations, *n* = 3. Statistical significance was detected by a two-tailed t-test. * * *P* < 0.01, * * * *P* < 0.001

In this study, we used the larval weight to evaluate soybean resistance to CCW. As shown in Table 1, the maximum and minimum values were 0.54 g and 0.09 g per larva in 2014 and 0.30 g and 0.01 g per larva in 2016, respectively. The ANOVA indicated that the resistance to CCW among the 121 accessions was significant (*P* < 0.001) in both years. Among the accessions, we identified two wild soybean lines (W11 and W99) that showed stable extreme performance in both years (Table 1). W99 was resistant to CCW, and the average weight of a single larva feeding on W99 was 0.06 g. In contrast, W11 was highly susceptible to CCW, and the average weight of a single larva feeding on W11 (0.39 g) was 6 times greater than that on W99. The evaluation of the antibiosis to CCW for 7 consecutive d also showed that the larvae feeding on W99 had a significantly lower weight than those feeding on W11 from 2 d to 7 d (Fig. 1). Thus, W11 and W99 were used to analyze the gene expression profiles of wild soybean lines after CCW attack.

**Table 1:** Descriptive statistics and ANOVA of the larval weight of the 121 accessions

Year	Population of 121 accessions					Accessions used in the RNA-Seq analysis	
	Max (g)	Min (g)	Mean ± SD (g)	CV (%)	G	Mean ± SD (g)-W11	Mean ± SD (g)-W99
2014	0.54	0.09	0.26 ± 0.09	34.37	***	0.54 ± 0.13	0.09 ± 0.02
2016	0.3	0.01	0.11 ± 0.05	50.69	***	0.23 ± 0.07	0.02 ± 0.01

*Max* maximum, *Min* minimum, *SD* standard deviation, *CV* coefficient of variation, *G* genotype, *W11* accession W11, and *W99* accession W99, *** *P*<0.001

Different lines are known to display different peak resistance times after insect attacks [15]. To confirm the peak resistance times of the two wild soybean lines, CCW-attacked and corresponding control leaves were harvested at seven time points (1 h, 6 h, 1 d, 2 d, 3 d, 5 d and 7 d) over a 7-d period. *LOX7* **(**glysoja_022393**)** is a lipoxygenase gene upstream of the JA signaling pathway that is up-regulated in leaves following wounding [16]. Vegetative storage protein β gene (*VSPβ*) (glysoja_036866) is located downstream of the defense network and is up-regulated after CCW attacks [5, 15]. Therefore, *LOX7* and *VSPβ* were chosen as early and late indicator genes, respectively.

**Fig. 2:**
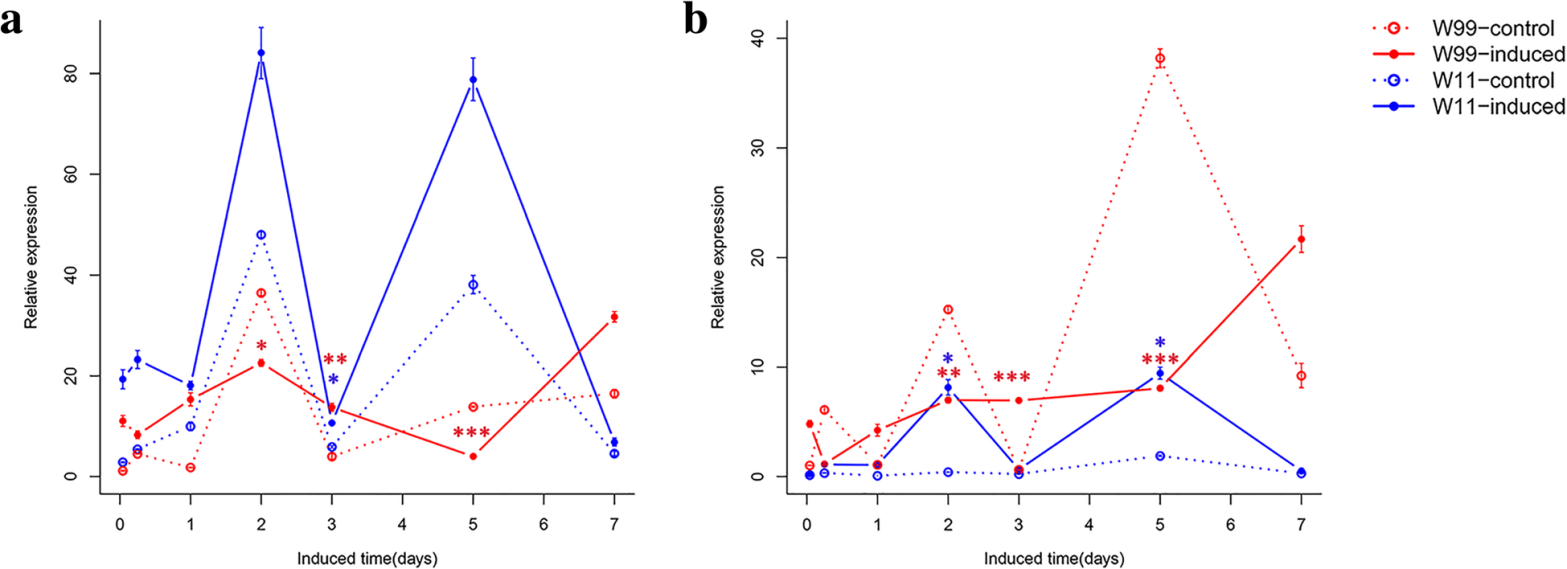
Dynamics of gene expression of the indicated genes *LOX7* (**a**) and *VSPβ* (**b**) in W11 and W99 before and after induction at seven time points (1 h, 6 h, 1 d, 2 d, 3 d, 5 d and 7 d). The constitutively expressed *tubulin* gene was used as a reference gene. The values were measured by the ΔΔCt method and compared with the control samples of W99 at timepoint 1 h. The error bars represent the standard deviations, *n* = 3. Statistical significance was detected by a two-tailed t-test. * *P* < 0.05, * * *P* < 0.01, * * * *P* < 0.001

The expression patterns of the two indicator genes between CCW-attacked and control plants at the same time point of both lines W11 and W99 were compared. The expression patterns of *LOX7* and *VSPβ* in W99 were similar. The expression of *LOX7* increased at 1 h, 6 h, 1 d, 3 d, and 7 d and weakened at 2 d and 5 d in CCW-attacked plants compared to control plants in W99 (Fig. 2a). The expression of *VSPβ* increased at 1 h, 1 d, 3 d, and 7 d and weakened at 6 h, 2 d, and 5 d in CCW-attacked plants compared to control plants in W99 (Fig. 2b). Among these time points, 3 d was the one at which *LOX7* and *VSPβ* exhibited the strongest response to CCW attack in W99. In W11, the expression of *LOX7* rapidly increased by 7-fold at 1 h after induction, and then the expression weakened and decreased to 4.2-fold at 6 h and approximately 2-fold at the later time points compared to control plants at the same time point (Fig. 2a). However, the expression of *VSPβ* was different and significantly induced at 2 d (20.1-fold) and 5 d (5.0-fold) after induction in CCW-attacked plants compared to control plants in W11 (Fig. 2b). To comprehensively evaluate the early and late network components of the defense response to CCW attacks, 1 d was chosen as a representative early time point in the resistant (R1d) and susceptible (S1d) lines based on *LOX7* expression differences between the two lines. For the late time point, Wang et al. reported that lines with different resistance levels showed different points of peak induced resistance [15], and for the induced expression profiles of two the indicative genes (Fig. 2), 2 d for W11 and 3 d for W99 were the peak points and were chosen as representative late time points in the susceptible (S2d) and resistant (R3d) lines, respectively.

### Significant DEGs in response to CCW feeding

The RNA-Seq clean data were aligned to the reference genome; approximately 37,000 genes were detected in each sample (Additional file 1: Table S1), and in total, 44,309 genes were detected. Among these genes, 3836 genes, including 3572 known genes and 264 predicted novel genes, were identified as non-redundant DEGs in all four comparisons (R1d, R3d, S1d, S2d) (Additional file 2: Table S2).

**Fig. 3:**
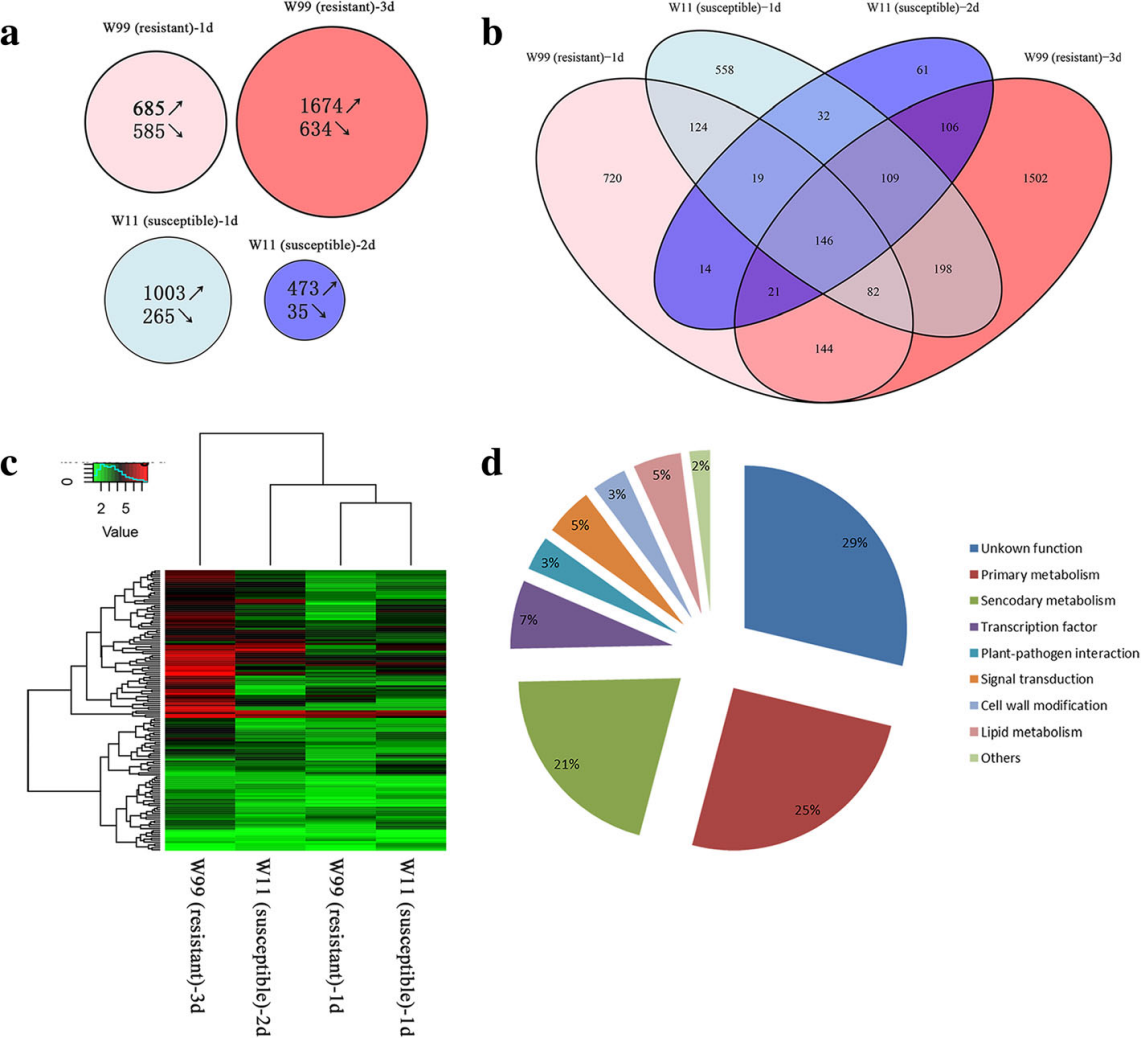
Characteristics of DEGs. **a** Venn diagram showing the number of up-regulated (upward arrow) and down-regulated (downward arrow) genes in the four comparisons identified by RNA-Seq; (**b**) Venn diagram showing the overlap of DEGs among the four comparisons; and (**c**) hierarchical clustering of 146 common DEGs in the four comparisons. The gene expression was quantified via RSEM v1.2.12 with the default options. The ‘value’ stands for log_2_-transformed expression of DEG. **d** Functional classification of the common DEGs. W11 (susceptible)-1d, W11 (susceptible)-2d, W99 (resistant)-1d, and W99 (resistant)-3d represent the comparison of CCW induction vs control in susceptible W11 at 1 d, susceptible W11 at 2 d, resistant W99 at 1 d, and resistant W99 at 3 d, respectively

In the susceptible line W11, 1003 up-regulated and 265 down-regulated DEGs were identified in CCW-attacked plants at 1 d after induction compared to control plants (Fig. 3a, Additional file 3: Table S3). In total, 473 up-regulated and 35 down-regulated DEGs were identified in CCW-attacked plants at 2 d after induction compared to control plants (Fig. 3a, Additional file 4: Table S4). In the resistant line W99, 685 up-regulated and 585 down-regulated DEGs were identified in CCW-attacked plants at 1 d after induction compared to control plants (Fig. 3a, Additional file 5: Table S5), whereas 1674 up-regulated and 634 down-regulated DEGs were identified in CCW-attacked plants at 3 d after induction compared to control plants (Fig. 3a, Additional file 6: Table S6). At the early time point, there were more up-regulated and fewer down-regulated DEGs in the susceptible line W11 than in the resistant line W99. However, at the late time point, the numbers of up-regulated and down-regulated DEGs were much lower in W11 than those in W99. In total, the greatest number of genes was responsive to the CCW attack in W99 at the late time point.

To better understand the response mechanism to CCW attacks in both wild soybean lines at different time points, GO term enrichment analyses were applied to the induced DEGs. The three GO categories were biological process (BP), cellular component (CC), and molecular function (MF). The GO terms with corrected *P*-values ≤0.01 are listed in Additional file 7: Table S7. Regarding BPs, the DEGs in W11 and W99 at the early time points (S1d and R1d) were significantly associated with the regulation of 3 biological processes and 3 molecular function processes. The functions of these DEGs were classified mainly as negative regulation. The DEGs in the susceptible line W11 at the late time point (S2d) were significantly enriched in the regulation of 23 biological processes, including the negative regulation of metabolic processes, cellular processes, proteolysis, peptidase activity, molecular function, secondary metabolic processes, responses to stress, etc. The DEGs in the resistant line W99 at the late time point (R3d) were significantly and specifically enriched in 3 photosynthesis processes and 2 secondary metabolic processes.

For CCs, the DEGs in R1d were significantly associated with the cell periphery and extracellular region. The DEGs in S2d and R3d were significantly enriched in some intracellular parts; 3 chromosome-related terms were associated with S2d, and 11 terms were associated with R3d, including chromosome, thylakoid photosystems in cells, etc.

Regarding MFs, the resistant line W99 expressed genes that were majorly associated with molecular function regulation at the early time point (R1d), whereas the genes were primarily associated with catalytic activity and binding at the late time point (R3d). The susceptible line expressed genes associated with molecular function regulation, catalytic activity and binding at the early time point (S1d), and the genes were mostly associated with molecular function regulation at the late time point (S2d).

KEGG analysis revealed the DEGs in both lines at early and late time points commonly enriched in the biosynthesis of secondary metabolites (Table 2). DEGs in R3d were enriched in 12 pathways, of which 5 were unique to the other three comparisons, including isoflavonoid biosynthesis, cyanoamino acid metabolism, starch and sucrose metabolism, circadian rhythm-plant, and photosynthesis-antenna protein. These results suggest that from the early to late time points after induction, compared with the susceptible line W11, the resistant line W99 enhanced its defense response by expressing more genes involved in defense processes.

**Table 2:** Enriched KEGG pathways of DEGs in the four comparisons and a common group

	Corrected *P*-value						
Pathway	R1d	R3d	S1d	S2d	146 Common DEGs	Pathways ID	Level
Biosynthesis of secondary metabolites	9.55E-03	1.64E-15	0.002504	2.68E-06	8.60E-04	ko01110	Global and overview maps
**Isoflavonoid biosynthesis**		1.67E-15				ko00943	Biosynthesis of other secondary metabolites
Flavonoid biosynthesis		2.09E-13	6.55E-04	7.50E-03		ko00941	Biosynthesis of other secondary metabolites
Phenylpropanoid biosynthesis		2.36E-06	2.50E-03	1.53E-04	3.54E-03	ko00940	Biosynthesis of other secondary metabolites
Metabolic pathways		9.83E-06		2.21E-04	3.54E-03	ko01100	Global and overview maps
Tryptophan metabolism		1.08E-06		1.46E-03	1.25E-03	ko00380	Amino acid metabolism
Glutathione metabolism		9.07E-08		7.87E-04		ko00480	Metabolism of other amino acids
**Cyanoamino acid metabolism**		9.35E-04				ko00460	Metabolism of other amino acids
**Starch and sucrose metabolism**		3.69E-04				ko00500	Carbohydrate metabolism
Cutin, suberin and wax biosynthesis		9.35E-04		3.16E-06		ko00073	Lipid metabolism
**Glycerolipid metabolism**		1.39E-03				ko00561	Lipid metabolism
**Photosynthesis-antenna proteins**		1.08E-06				ko00196	Energy metabolism
Ribosome biogenesis in eukaryotes			2.50E-03			ko03008	Translation

Bold font indicates that the 5 groups are only significantly different in R3d. R1d, R3d, S1d, and S2d represent the comparison of the treatments vs controls of W99 at 1 d, W99 at 3 d, W11 at 1 d, and W11 at 2 d. KEGG: Kyoto Encyclopedia of Genes and Genomes. DEG: differentially expressed gene

### Common and unique regulated genes in the two lines at both early and late time points

In total, 146 common DEGs up-regulated in all four comparisons were identified, constituting the basic defense network at both time points in both lines (Fig. 3b, Additional file 8: Table S8). The expression levels of 82 common genes (56%) were enhanced by 1.86-fold on average from 1 d to 2 d after induction in W11, while the expression levels of 127 common genes (87%) were enhanced by 3.51-fold on average from 1 d to 3 d after induction in W99 (Fig. 3c). We then consolidated the 146 common DEGs into 9 categories according to their putative function (Fig. 3d). The major categories were unknown function (28%) and primary metabolism (25%). The secondary metabolism category also accounted for a large portion (21%), including 10 protease inhibitors and 11 phenylpropanoid genes. The proportion of TFs, plant-pathogen interactions, signal transduction, cell wall modifications and lipid metabolism ranged from 3 to 7% (Fig. 3d). Moreover, the GO analysis revealed that these genes were enriched in the regulation of biological processes and molecular functions. KEGG analysis revealed that these genes were enriched in the biosynthesis of secondary metabolites, tryptophan metabolism, phenylpropanoid biosynthesis and metabolic pathways. The enriched GO terms and KEGG pathways in the common DEGs were consistent with the results of separate analyses of R1d, R3d, S1d and S2d (Table 2, Additional file 7: Table S7).

Except for the common DEGs, every comparison had unique DEGs whose expression was not significantly induced in the other three comparisons, which indicated their specific induced resistance. Among the four comparisons, 18.8, 39.2, 14.5 and 1.6% of the non-redundant DEGs were uniquely observed in R1d, R3d, S1d and S2d, respectively (Fig. 3b). GO and KEGG analyses were applied to unique DEGs. The unique DEGs of R1d and S2d were enriched in 2 and 3 catalytic activity processes, respectively. Moreover, the unique DEGs in R3d were enriched in 9 terms concerning BPs, including secondary metabolic processes and photosynthesis processes; 7 terms were enriched in MFs, including binding and catalytic activity; and 13 terms were enriched in CCs, primarily intracellular parts (Additional file 9: Table S9). Similar results were observed from the KEGG enrichment analysis: unique DEGs of S1d were enriched in ribosome biogenesis in eukaryotes and plant hormone signal transduction, and unique DEGs of R3d were enriched in 9 pathways, including secondary metabolites, isoflavonoid biosynthesis, photosynthesis-antenna protein metabolism and so on (Additional file 9: Table S9). Interestingly, except for some known resistance processes, the photosynthesis process was enriched in the unique DEGs of R3d. Further analysis revealed that the photosynthesis-related genes were all down-regulated (Additional file 6: Table S6). The results also indicated that from the early to late time points after induction, the resistant line W99 enhanced the defense response in terms of both common and unique defense aspects; however, this did not occur in the susceptible line W11.

### CCW attack stimulated some defense-related molecules and pathways in both lines

Among the 3836 non-redundant DEGs, abundant transcripts were up-regulated, including some bona fide defense molecules and pathways (Additional file 2: Table S2). The expression of the *PR5* gene (gene ID: 100037479), the repetitive proline-rich protein *PRP2* gene (gene ID: 547830), the LRR protein-related protein gene (gene ID: 100305447), the LRR receptor-like serine gene (gene ID: 100813275), the disease resistance protein *RPS2* gene (gene ID: 100793054), and the vegetative storage protein β *VSPβ* gene (gene ID: 547820) was up-regulated in all four comparisons. In addition, secondary metabolism played an important role in response to herbivore induction. The expression of genes encoding protease inhibitors (gene ID: 100170690, 100170748, 100527215, 100170691, etc.), laccase (gene ID: 100799343, 100797234, 100798416, 100796956, 100797749, 100812336), peroxidase (gene ID: 100803637, 100810392, 100790279, 100803503, etc.) and NADPH: isoflavone reductase (*N:IFR*, gene ID: 547659) was found to be up-regulated. Further, genes associated with the JA pathway were stimulated, including those encoding the TF MYC2 (gene ID: 100809422, 100794354, 100816551, 100807098, 100809888, 100798018, 100795524), phospholipase (gene ID: 100802647, 100777122, 100798569, 100778146, 100795779, 547865), lipoxygenase (gene ID: 547836, 100814036, 547694, 547835, 100814410, 100803358, etc.), allene oxide synthase (gene ID: 100037481, 100807192), allene oxide cyclase (gene ID: 100803666, 100805194), 12-oxophytodienoic acid reductase (gene ID: 100818208, 100797662, 100816003, 100796758), oxalate-CoA ligase (gene ID: 100779306), acetyl-CoA acyltransferase (gene ID: 100789930), jasmonate O-methyltransferase (gene ID: 102660621 and 100783664), and jasmonate ZIM domain-containing proteins (gene ID: 100801449). Most up-regulated genes involved in the JA pathway were found in the resistant line (Additional file 5: Table S5, Additional file 6: Table S6).

### CCW attack repressed some growth-related transcripts in both lines

Except for numerous up-regulated DEGs, the expression of more than 1000 non-redundant DEGs was down-regulated (Additional file 2: Table S2) in one or more comparisons of the 3836 non-redundant DEGs. Among the repressed transcripts, those involved in photosynthesis were abundant, such as those involved in ATP synthesis (gene ID: BGI_novel_G000002), photosystem I (gene ID: 100809697, 100306036, 100500098, 100305746, 100806824), photosystem II (gene ID: 100306223, 100305487, 102669614) and light harvesting (gene ID: BGI_novel_G000242, 100819580, BGI_novel_G000851, 100793702, 100790960, 100800351, 100779387, 547819). Additionally, the expression of genes related to nutrient metabolism, including phosphate transporter *PT1* (gene ID: 100802890) and *PT2* (gene ID: 100792177), and genes encoding sulfate transporters (gene ID: 100781327, 100791091, 100798723, 100804670, and 100780331) and thioredoxin (gene ID: 100305773), was down-regulated. Furthermore, 28 down-regulated genes (26 in W99 and 2 in W11), such as those encoding the auxin influx carrier *LAX3* (gene ID: 100778463), PIN auxin efflux transporters (gene ID: BGI_novel_G001247, 100784879, and 100789817), auxin-responsive proteins (gene ID: 100791342, 547965, 100305535, etc.), and auxin response factors (gene ID: 100805456, 100804628, 100780393, and 100802387), were related to auxin.

### Significantly differentially expressed TFs in response to CCW feeding in both lines

**Fig. 4:**
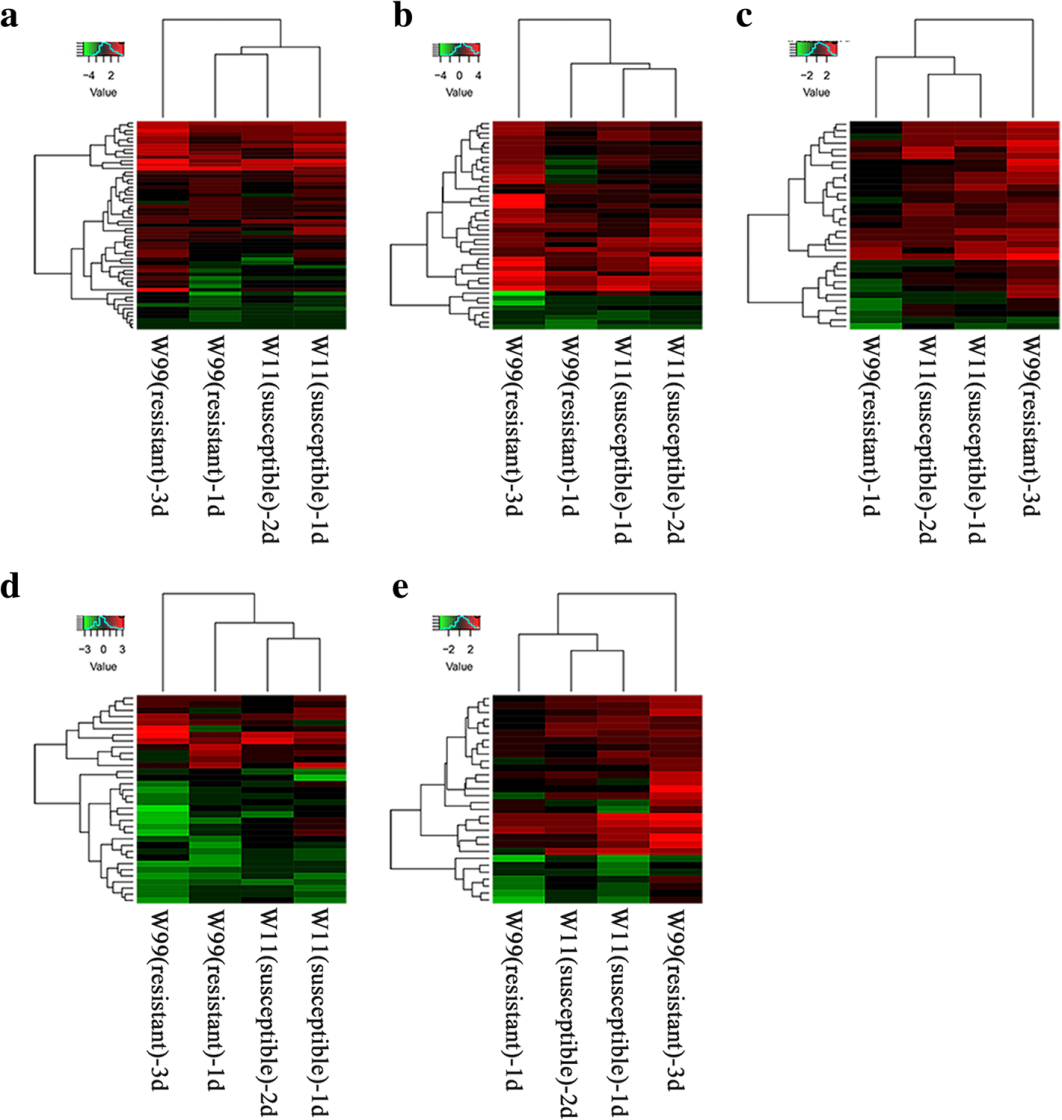
Hierarchical clustering analysis of differentially expressed TFs. **a** ERF family; (**b**) MYB family; (**c**) WRKY family; (**d**) bHLH family; and (**e**) NAC family. The gene expression was quantified via RSEM v1.2.12 with the default options. The ‘value’ stands for log_2_-transformed expression of DEG. W11 (susceptible)-1d, W11 (susceptible)-2d, W99 (resistant)-1d, and W99 (resistant)-3d represent the comparison of CCW induction vs control in susceptible W11 at 1 d, susceptible W11 at 2 d, resistant W99 at 1d, and resistant W99 at 3 d, respectively

TFs that regulate gene expression by binding to cis-regulatory elements of target genes in a sequence-specific manner play an important role in gene regulatory networks. We identified 91, 185, 111 and 48 significantly expressed TFs in R1d, R3d, S1d and S2d, respectively (Fig. 4, Additional file 10: Table S10). In total, 305 non-redundant TFs belonging to 34 TF families, including 57 ERFs, 46 MYBs, 34 WRKYs, 34 bHLHs, 31 NACs, 4 ARFs, 2 TCPs, etc., were identified at the early- and late-induction time points in both accessions. Among them, 10 TFs, including 6 ERFs, 1 C_2_H_2_, 1 MYB, 1 WRKY and 1 CPP, were commonly regulated in the four comparisons. We performed a hierarchical clustering of ERFs, MYBs, WRKYs, bHLHs, and NACs (Fig. 4). As shown in Fig. 4, most ERF, MYB, WRKY and NAC TFs were up-regulated after induction, and most bHLH TFs were down-regulated. Regardless of whether the TFs were up-regulated or down-regulated, TF expression was the highest at 3 d after induction in the resistant line W99. Furthermore, TFs at 3 d after induction in W99 clustered away from those in the other three comparisons. We then constructed a phylogenetic tree consisting of five TF families (Additional file 11: Figure S1); most up-regulated TFs clustered into the same groups away from the down-regulated groups. These results were consistent with previous findings in that most genes with similar functions tended to cluster together [17].

To discover herbivore-induced gene regulatory networks in wild soybean, we further analyzed the TF binding sites in the promoters of 3 up-regulated defense-related genes (*LOX*: 100803358, *VSPβ*: 547820, *PR5*: 100037479) and 3 down-regulated growth-related genes (*PIN*: 100784879, *PT1*: 100802890, *PSI*: 100806824) (Additional file 2: Table S2). Six promoter sequences were successfully retrieved from the *Glycine soja* genome, and the obtained sequences were put into the Plant Transcription Factor Database. Fifty-three binding sites of 28 co-expressed TFs were identified from 6 input sequences (Additional file 12: Table S11). The defense-related genes were predicted to bind major MYBs, NACs and WRKYs. *WRKY53* (732586), *WRKY43* (100796481), *WRKY22* (100815829), *WRKY28* (100816891), a MYB TF (100791349) and a CPP TF (100804447) were predicted to bind to all 3 defense-related genes. In contrast to the defense-related genes, the growth-related genes were predicted to bind fewer co-expressed TFs. Two zinc finger TFs (*ZAT10*: 100776038; *ZAT10-like*: 100800095), 2 R2R3-MYB TFs (*MYB29A2*: 100101837; *MYB29B2*: 100807569) and a trihelix TF (*GT-3b-like*: 100794230) were predicted to bind with the *PIN* auxin efflux transporter. A zinc finger TF (*ZAT10-like*: 100800095) and a CPP TF (100804447) were observed to bind with *PT1*. In wheat, *TaZAT8* increased the transcripts of *NtPT1* and *NtPT2* in overexpression transgenic tobacco plants [18]. In our study, the expression of the *ZAT10-like* TF was repressed at 1 d but stimulated at 3 d after induction, and the expression of *PT1* was repressed at 1 d and recovered to non-significant levels at 3 d after induction. A MADS TF (100809514) and an SBP TF (100787416) were observed to bind with *PSI*.

### Validation of RNA-Seq data

The average correlation coefficient between any two biological replicates was 97.2% (Additional file 13: Table S12), indicating stable consistency among the biological replicates. A hierarchical clustering analysis showed that the three biological replicates of the four comparisons all clustered together with their own DEGs, indicating that our experimental design was reliable (Additional file 14: Figure S2).

To confirm the results of the RNA-Seq analysis, 30 DEGs between 2 comparisons or among the four comparisons were selected for qRT-PCR. Twenty-two genes exhibited good PCR amplification efficiency and were selected for further validation (Additional file 15: Table S13). We then performed a correlation analysis of the RNA-Seq and qRT-PCR expression data for the resistant (W99) and susceptible (W11) lines. The Pearson correlation coefficients were 0.937 (*P* = 2.2e-16) and 0.952 (*P* = 2.2e-16) for the resistant (W99) and susceptible (W11) line, respectively (Additional file 16: Figure S3). These results indicated that the data from RNA-Seq experiments were reliable.

**Fig. 5:**
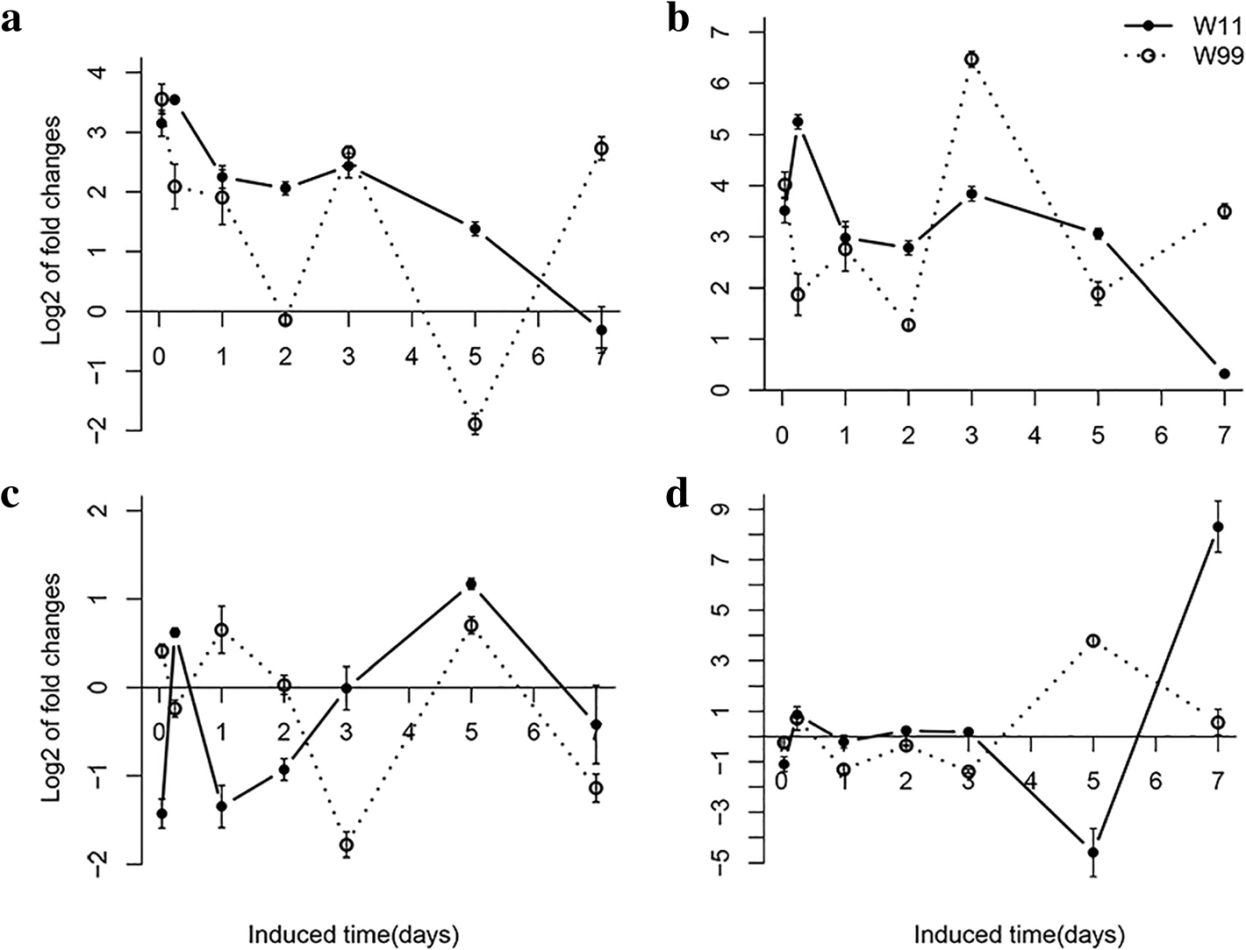
Expression patterns of *LOX* (**a**), *PRP2* (**b**), *PSI* (**c**), and *PT1*(**d**) at seven time points (1 h, 6 h, 1 d, 2 d, 3 d, 5 d and 7 d) after induction in resistant (W99) and susceptible (W11) lines by qRT-PCR analysis. The constitutively expressed *tubulin* gene was used as a reference gene. The values were measured by the ΔΔCt method and compared with those of the controls at the same time points. The values are expressed as log_2_-fold changes. > 0 indicates up-regulated, < 0 indicates down-regulated, and 0 indicates unchanged. The error bars represent the standard deviations, *n* = 3

To obtain the detailed spatial expression patterns of the DEGs in the two lines, we selected 2 up-regulated defense-related genes (*LOX*: 100803358 and *PRP2*: 547830) and 2 down-regulated growth-related genes (*PSI*: 100806824 and *PT1*: 100802890) of 22 and analyzed their expression at seven time points. The expression of *LOX* and *PRP2* in W11 was immediately enhanced at 1 h and peaked at 6 h, after which the expression weakened and returned to basal levels at 7 d compared to the corresponding control plants. However, the up-regulated genes expression in W99 peaked at multiple time points (1 h, 1 d, 3 d and 7 d) after induction compared to the corresponding control plants (Fig. 5a and b). The expression patterns were similar to those of *LOX7* and *VSPβ* (Fig. 2). The different patterns of the genes in the two lines indicated that the regulatory mechanism might be more complicated in the resistant line W99 than in the susceptible line W11. Regarding the down-regulated genes, the expression of *PSI* was repressed at 1 h and 1 d after induction in W11 and repressed at 3 d and 5 d after induction in W99 compared to the corresponding control plants (Fig. 5c). The expression of the *PT1* gene was down-regulated at 1 d and 3 d but up-regulated at 5 d after induction compared to the corresponding control plants in W99. However, the expression did not significantly change during the first 3 d and was repressed at 5 d after induction compared to the corresponding control plants in W11 (Fig. 5d). In the resistant line, the expression of two growth-related genes was down-regulated at the time points at which the expression of defense-related genes was strongly up-regulated, which could be due to saving enough energy for the defense system. The phenomenon was not obvious in the susceptible line.

### Transgenic soybean plants overexpressing *GmPT1* were susceptible to CCW

In a previous study, resistance provided by some up-regulated defense-related genes, such as *GmVSPβ* and *GmN:IFR,* was identified in transgenic plants [19]. However, to the best of our knowledge, no growth-related genes significantly down-regulated after CCW attacks have been characterized for their role in resistance. In this study, a major growth-related gene involved in photosynthesis, nutrient metabolism, auxin metabolism, etc. was down-regulated after induction. To test the role of growth-related genes in resistance to CCW, we chose phosphate transporter *PT1* (100802890) for characterization.

**Fig. 6:**
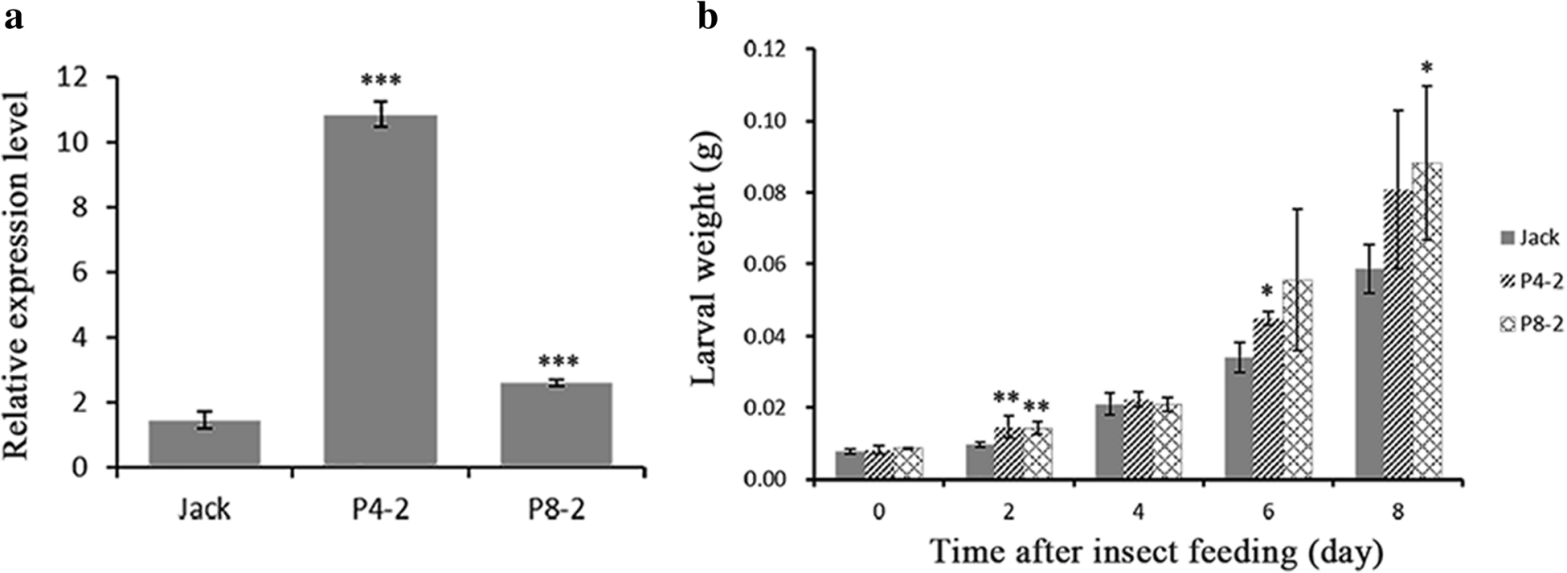
Expression level and herbivore resistance of *GmPT1* transgenic soybeans. **a** Results of a qRT-PCR analysis of *GmPT1* gene expression in transgenic plants. The constitutively expressed *tubulin* gene was used as a reference gene, and the values were measured by the ΔΔCt method and compared with the control line Jack. **b** Dynamic changes in CCW larval weight feeding on control and transgenic plants for 8 consecutive d. The error bars represent the standard deviations, *n* = 3. Statistical significance was detected by a two-tailed t-test. * *P* < 0.05, * * *P* < 0.01, * * * *P* < 0.001

The amino acid sequence of *PT1* in W05 (glysoja_000733) was completely consistent with the sequence of *PT1* (**GenBank: FJ797401.1**) in Williams 82 reference. The results indicated that the sequence of *PT1* was highly conserved in wild soybean and cultivated soybean. To characterize the function of *PT1* in soybean resistance to CCW, we developed two independent *GmPT1* transgenic soybean lines, P4–2 and P8–2 (T_4_), by *Agrobacterium*-mediated transformation (Additional file 17: Figure S4). The expression of *GmPT1* in the transgenic plants was analyzed by qRT-PCR (Fig. 6a). Compared with the non-transgenic controls, both P4–2 and P8–2 showed high *GmPT1* expression levels. The resistance levels of P4–2 and P8–2 were evaluated in a force-feeding experiment. The mean larval weight of CCWs feeding on the P4–2 plants was significantly higher than that feeding on the control plants at 2 d and 6 d, and the P8–2 line showed significant resistance at 2 d and 8 d (Fig. 6b). These results showed that *GmPT1* negatively regulated soybean resistance to CCW.

## Discussion

### The resistant wild line regulated a more complicated network response to induction

Lines W99 and W11 strongly differed in their constitutive resistance to CCW (Fig. 1, Table 1). After induction, they also showed different responses. The resistant line W99 expressed 1270 and 2308 DEGs that were significantly associated with 1 and 12 pathways at early and late time points, respectively. The susceptible line W11 expressed 1268 and 508 DEGs that were significantly associated with 4 and 7 pathways at early and late time points, respectively (Fig. 3a, Table 2). Both the number of DEGs and the number of pathways clearly increased with increasing induction time in the resistant line W99. However, although the susceptible line W11 initiated a defense response as strong as that of the resistant line W99 at the early time point, the number of DEGs did not increase at 2 d after induction. In addition, excluding 146 common DEGs, the resistant line expressed more unique DEGs (18.8% for R1d and 39.2% for R3d) than did the susceptible line (14.5% for S1d and 1.6% for S2d) (Fig. 3b). The number of TFs in W99 (276 TFs) was also greater than that in W11 (159 TFs). Thus, both the number of induced genes and the degree of defense response were stronger in the resistant line W99 than in the susceptible line W11.

In addition, the two wild soybean lines used in the experiment differed not only in their resistance to herbivores but also in their morphology (Additional file 18: Figure S5). A phylogenetic analysis of 367 lines, including 105 wild and 262 cultivated soybeans, classified W11 into group V along with cultivated soybeans [20]. In the present study, the genomic sequence of W11 had a higher similarity to the genomic sequence of Williams 82 than to that of W99 (Additional file 19: Figure S6a, b), and fewer SNPs were aligned (Additional file 19: Figure S6c), which was consistent with the phylogenetic analysis results [20]. A similar phenomenon resulted from the phylogenetic analysis of 25-resequenced soybean lines in which two wild lines showed an admixture with landraces and elite cultivars [11]. The wild lines that were relatively closely related to cultivars may represent a bridge in soybean domestication. Domestication could severely reduce the genetic diversity, half of which is related to resistance [21, 22]. Therefore, the sequence difference between W11 and W99 may be one reason for the difference in their resistance to CCW. Moreover, the differences in gene sequence may lead to differences in metabolite formation. Kim et al. compared the genomes of wild and cultivated soybeans and identified approximately 2.5 million SNPs, including 38,598 non-synonymous SNPs in coding sequences, and 196,356 InDels, including 2398 InDels in coding sequences causing frameshifts, in 2235 genes. The fraction of nucleotide sequence differences played a role in the functional consequences between the wild and cultivated soybeans [23]. Some genes whose functional consequences were determined by the difference in the gene structure were identified. A soybean pan-genome analysis identified that the homologs of senescence-associated gene 101 (*SAG101*) with amino acid changes are candidate genes that affect the oil content and confer resistance to *Phytophthora sojae* [24]. Lu et al. identified that phosphatase 2C-1 (PP2C-1) from wild soybean contributes to the increase in seed weight/size [25]. In contrast, the PP2C-2 allele with variations in a few amino acids at the N-terminus did not exhibit this function. Therefore, a certain level of genetic diversity in the gene sequence between W11 and W99 might result in differences in metabolite formation, especially for metabolites related to insect resistance, which may provide another explanation for the two materials with different resistance to CCW in our study.

The time points used in this study were chosen at the peaks of induced resistance as reported by Wang et al. [15]. The early time point was same for both lines, occurring at 1 d, and the late time point differed, occurring at 2 d for W11 and 3 d for W99. However, the limitation of this design is that some information related to resistance could have been missed. To comprehensively understand the genetic mechanism of soybean resistance to CCW, additional samples at the same time points need to be considered in the future.

### Defense-related gene response to CCW attack

CCW typically induces genes constituting the basic defense system. In our study, ten protease inhibitors belonging to the Kunitz and Bownman-Birk families were identified as being encoded by up-regulated genes (Additional file 8: Table S8). Protease inhibitors are present in all organisms and are well known to participate in defense against pests and pathogens [26–28]. Among them, *glysoja_000750* is homologous to *GmPPI-1*, which is induced in soybean roots infected with soybean cyst nematode [29]. Eleven genes associated with the phenylpropanoid pathway, which governs the largest group of secondary metabolite responses to stresses, were significantly regulated. Phenylpropanoid biosynthesis has been confirmed to respond to biotic and abiotic stresses in other studies [30–32]. Five genes associated with cell wall metabolism were induced, and the cell wall provides a physical defense against insect feeding. Similar results were also found in *Arabidopsis*, maize, and rice [33–35]. Seven genes associated with lipid metabolism, including allene oxide synthase-like and linoleate 9S-lipoxygenase, are typically up-regulated; these genes are associated with JA biosynthesis, which play an important role in phytohormone regulation in insect resistance [36, 37]. Five genes function in plant-pathogen interactions, including 2 genes encoding the LRR and PR5 proteins. Ten commonly regulated TFs, including 6 ERFs, 1 C_2_H_2_, 1 MYB, 1 WRKY and 1 CPP, as well as ERFs, MYBs and WRKYs were shown to be associated with methyl jasmonate (MeJA)-responsive or insect-induced gene regulatory networks in previous studies involving cultivated soybean and *Arabidopsis* [4, 5]. The identified genes in our study provided an abundant resource for the breeding of insect-resistant soybeans.

### CCW attack down-regulated growth-related genes in wild lines

In response to the CCW attack, abundant genes were up-regulated and numerous genes were suppressed in the two wild soybean lines. A greater proportion of genes (34.1%) were down-regulated in the resistant line than in the susceptible line (16.9%). Among these genes, 6 photosynthesis-associated genes were down-regulated in the susceptible line and 31 photosynthesis-associated genes were down-regulated in the resistant line (Additional file 3: Table S3, Additional file 4: Table S4, Additional file 5: Table S5, Additional file 6: Table S6). Similar results have been reported as follows: a series of photosynthesis-related genes were down-regulated in cotton bolls, kiwifruit, *Nicotiana attenuata* and tomato after bollworm infestation, other insect infestation, *Manduca sexta* infestation and whitefly herbivory [38–41]. In addition, two phosphate transporter genes (*PT1* and *PT2*) were significantly down-regulated after induction. A similar result was found in cotton, and some transcripts related to phosphate transporter and phosphate synthesis were down-regulated after bollworm infestation [38]. Phytohormones regulate almost all aspects of plants during their lifecycle. After CCW attacks, substantial numbers of transcripts associated with JA were up-regulated, and quite a few transcripts associated with auxin, which is usually defined as a growth-related phytohormone, were down-regulated. In chickpea, growth-associated hormone pathways such as those of gibberellic acid (GA) and auxin were also suppressed after insect attacks [42]. To cope with challenges as efficiently as possible, plants will up-regulate their sink metabolism to satisfy the energy requirements of the activation of defense reactions, and simultaneously, growth-related genes will be down-regulated to balance metabolism [43–45]. In our study, the resistant line W99 had the greatest defense response at 3 d after induction and had the greatest extent of down-regulation of growth-related genes. This result was consistent with the balance of metabolism.

### *PT1* is sensitive to CCW

In this study, two transgenic soybean lines overexpressing *GmPT1* both showed significant susceptibility to CCW (Fig. 6). Interestingly, the QTL relation to the larval weight of CCW on Gm10 (LG-O) overlapped with the cis-eQTL *qPO.1* for *GmPT1* in a recombinant inbred line (RIL) population derived from the cross between Kefeng No. 1 and Nannong 1138–2 [46, 47]. The *GmPT1* gene was also located in the region of the same QTL [46]. The expression levels of *GmPT1* were markedly up-regulated in soybean under Pi starvation. P acquisition efficiency (PAE) and internal P-use efficiency (PUE) increased in *GmPT1* transgenic tobacco plants [46]. In *Arabidopsis*, the Pi-deficient mutant *pho1* exhibited increased resistance to insect herbivores, and JA-related genes were induced and JA levels were high; wild-type plants activated JA-related genes and increased JA and JA-Ile levels in shoots and roots under Pi starvation. Moreover, wild-type *Arabidopsis*, as well as *Nicotiana benthamiana* and tomato, was more resistant to herbivores under Pi-deficient conditions than under Pi-sufficient conditions [48]. The results of the study indicated that the increased resistance to herbivores may result from the induction of JA. In other studies of *Brachypodium distachyon*, soybean and *Arabidopsis*, JA signaling-related genes were also significantly regulated in response to Pi starvation [49–51]. In addition, Hassel et al. reported that an *Atpht4;6* knockout mutant exhibited reduced growth, altered cell wall composition, and increased levels of both salicylic acid (SA) and resistance against the virulent *Pseudomonas syringae* strain DC3000 [52]. The results of the present study suggested that plants could increase their resistance to biotic stress via phytohormone signaling under Pi starvation.

Sham et al. reported that defense responses controlling biotic and abiotic stress may interact synergistically or antagonistically in response to multiple stresses in the natural environment [53]. Insects and low Pi in the soil are two major factors limiting soybean yield and seed quality. Research on the relationship between soybean resistance to insects and P use is important for reducing the application of pesticides and fertilizers during soybean cultivation. Additional research on the *PT1* gene may provide new insights into this relationship.

### Difference in the response to CCW attacks between wild and cultivated soybean lines

Using RNA-Seq and microarray analysis, Wang et al. revealed the transcriptome profiles of two soybean cultivated lines, WX (R) and NN (S), at peak time points after induced resistance [5, 15]. In the present study, in addition to the peak time points, we added a common time point (1 d) according to the different expression patterns of *LOX7* and *VSPβ* (Fig. 2). At the early time point, the expression of *VSPβ* increased in the susceptible line W11. At the same time, the expression of *LOX7* decreased in the susceptible line W11. However, the expression of two genes reached a small apex in the resistant line W99 at 1 d after CCW attack. Thus, 1 d was chosen as the early-induction time point for both wild lines, whereas 2 d for W11 and 3 d for W99 were chosen as late-induction time points. Overall, the response of the susceptible line to CCW was shorter than that of the resistant line in both the wild and cultivated lines. As the RNA-Seq time points increased, we identified 3836 non-redundant DEGs (Additional file 2: Table S2), which was much more than the number of non-redundant DEGs in the cultivated lines (1096 from microarray and 2405 from RNA-Seq analyses) [5, 15]. A microarray analysis revealed 100 down-regulated transcripts in the susceptible line NN [15]. Two hundred and sixteen and 698 down-regulated DEGs were identified in the resistant lines WX and NN after the CCW attack via RNA-Seq analysis, respectively [5]. In wild soybean, 585 and 634 DEGs were down-regulated in the resistant line W99 at 1 d and 3 d, respectively (Fig. 3a), and 265 and 35 DEGs were down-regulated in the susceptible line W11 at 1 d and 2 d, respectively (Fig. 3a). Therefore, fewer DEGs were down-regulated in the resistant cultivated soybean line than in the susceptible line. However, more DEGs were down-regulated in the resistant wild line than in the susceptible line. We speculated that the wild soybean lines had to down-regulate numerous genes that were unrelated to resistance pathways to meet the demand for insect resistance because, in contrast to cultivated soybean, these lines probably lack strong primary metabolism as a result of artificial selection and breeding. Moreover, the down-regulated DEGs in R3d were enriched in photosynthesis terms; however, these terms were not enriched in the cultivated lines (Additional file 6: Table S6).

TFs play important roles in the response to environmental stresses. Based on our analysis, 305 non-redundant TFs were identified; however, based on the analysis of the two cultivated lines, only 45 TFs were identified in the resistant line WX [15]. Among those TFs, WRKYs, MYBs, NACs and bHLHs were typically identified in wild soybean and cultivated soybean. ERFs and TCPs were specifically significantly expressed in the wild lines. The ERF family has been identified and implicated in many diverse functions involved in cellular processes, such as hormone signal transduction and responses to biotic and abiotic stresses [54]. TCPs are members of a plant-specific gene family and play important roles in the evolution and developmental control of plant forms. A previous study also showed that ERF and TCP families were also regulated in response to MeJA in *Arabidopsis* [4].

In total, compared with the cultivated lines, the wild lines exhibited a different defense response to the CCW attack. Additional research is needed to elucidate the mechanism of wild soybean defense against insects.

## Conclusion

Wild soybean houses a gene pool from which breeders can discover new elite genes. Here, the transcriptomes of a constitutively CCW-resistant line and a CCW-susceptible line were analyzed at early- and late-induction time points. In total, 3836 non-redundant DEGs, including 305 TFs, were significantly expressed after induction. The resistant line exhibited a longer and stronger defense response than the susceptible line. Further analysis revealed that the induction of CCW not only up-regulated defense-related genes, including jasmonic acid (JA)-related genes, plant-pathogen related genes, and genes encoding protease inhibitors, but also down-regulated growth-related genes involved in photosynthesis, nutrition metabolism, and auxin metabolism. The phosphate transporter *PT1*, which is a representative growth-related gene, was transformed into soybean; the transgenic soybean plants were susceptible to CCW. These results described transcriptome reprograming after herbivore induction in wild soybean, identified the susceptibility of growth-related genes, and provided new resources for the breeding of herbivore-resistant cultivated soybean.

## Methods

### Plant materials, field growth and insect bioassay

All 121 wild accessions used in this study were provided by the National Center for Soybean Improvement (Nanjing, China) [20]. We evaluated the CCW resistance of the populations over 2 years (2014 and 2016) at the Jiangpu Experimental Station of Nanjing Agricultural University (Nanjing, China). The field experiment and insect bioassay were performed as previously reported by Rector et al. [55]. The materials were grown in hill plots in a randomized complete block design with three replications. Each replication contained 4 hills with 20 plants from each accession. The hills were planted every 50 cm in rows that were spaced 50 cm apart for the same material and every 100 cm in rows spaced 100 cm apart between materials. A bamboo pole was placed close to each hill to support the twinning stems. The field was surrounded by a nylon mesh to exclude hares and other animals, and no chemical insecticides were used throughout the soybean growth period. Approximately 45 d after germination, the upper fully expanded leaves were selected for the bioassay, which was performed in the laboratory.

To evaluate the resistance of the wild soybean population, force-feeding experiments were conducted as previously described by Fan et al. [56]. The CCW larvae were maintained year-round on an artificial diet (Institute of Plant Protection, Jiangsu Academy of Agricultural Sciences, Nanjing, China). Five 2-instar CCW larvae were raised in a culture tank with a fresh leaf for seven d, and the fresh leaves were replaced every 2 d. The larvae were weighed on the seventh day after feeding. The bioassays were replicated in the same manner as the field experiments. Microsoft Excel 2010 was used to statistically analyze the data.

To evaluate the resistance of the wild soybean lines W11 and W99 and the *GmPT1* transgenic soybean lines to CCW, two additional force-feeding experiments were conducted. One experiment included wild soybean lines W11 and W99; the other involved in non-transgenic control (Jack) and *GmPT1* transgenic soybean lines. Five 2-instar CCW larvae were raised in a culture tank with a fresh leaf for seven d or eight d. For W11 and W99, the fresh leaves were replaced at 1, 2, 3, 5 and 7 d after feeding. For the *GmPT1* transgenic soybean and control lines, the fresh leaves were replaced every 2 days. The larvae were weighed before feeding and when the leaves were replaced. Each experiment was repeated three times. Microsoft Excel 2010 was used to statistically analyze the data.

### Plant growth, treatment and harvest in the greenhouse

Soybean seedlings were sown in 10 cm-diameter plastic pots that contained nutrient-enriched soil and vermiculite at a ratio of 1:1. After germination, seedlings that presented similar growth statuses were included in the experiment. The seedlings were grown in a greenhouse whose conditions included a 28 °C/26 °C (day/night) temperature, a 16 h/8 h (day/night) photoperiod and 70 ± 10% relative humidity.

Three weeks after germination, two third-instar CCW larvae were fixed on the third trifoliate leaves of the W99 and W11 wild lines via white net bags for 2 h; subsequently, the larvae were removed, and the induction time started. Control plants were not exposed to CCWs. The leaves of the control and treated plants were harvested at seven time points: 1 h, 6 h, 1 d, 2 d, 3 d, 5 d, and 7 d after induction.

For transcriptome analyses and qRT-PCR experiments, the induced leaves of the treated plants and the corresponding leaves of the control plants were collected at the seven sampling times from both the W11 and W99 lines; each line included three biological replicates. All samples were stored at − 80 °C.

### Total RNA isolation and qRT-PCR

The total RNA of 84 leaf samples was extracted in accordance with the manufacturer’s instructions with the use of a plant RNA extraction kit (TianGen, Beijing, China). Approximately 1 μg of RNA was then used to develop a 20 μl system to synthesize cDNA via a PrimeScript RT Reagent Kit with gDNA Eraser (TaKaRa, Japan). The constitutively expressed *tubulin* gene (*glysoja_029479*) was used as a reference gene for qRT-PCR; each sample was measured in three technical replicates. qRT-PCR was conducted on an ABI 7500 real-time PCR system (Applied Biosystems, Foster City, CA, USA) with SYBR Premix ExTaqII (TaKaRa, Japan). The Sequence Detection System (SDS) software v.1.4 of the ABI 7500 system was used to analyze the data. The amplification efficiencies of the primers were calculated according to the equation E = 10^[− 1/slope]^ [57], and the relative expression level was analyzed by the ΔΔCt program within SDS v.1.4. Information concerning all qRT-PCR primers is provided in Additional file 15: Table S13.

### RNA-Seq experimental design and data analysis

The CCW-attacked and corresponding control samples of W11 at 1 d and 2 d after induction and the CCW-attacked and corresponding control samples of W99 at 1 d and 3 d after induction were chosen for RNA-Seq experiments. Three biological replicates were used for each treatment, and there were a total of 24 samples (Additional file 1: Table S1). The extracted total RNA was quantitated via a NanoDrop spectrophotometer, and the RNA integrity number (RIN) was measured with an Agilent Bioanalyzer. After qualification, one μg of RNA was used for library construction in accordance with the protocol of an Illumina TruSeq RNA Sample Preparation system. The sequences of the RNA libraries were measured via an Illumina HiSeqX platform in conjunction with paired-end 2 × 150 bp. The average data yield for each sample was 6.69 Gb of total raw reads, and the percentage of Q30 bases was > 85%.

The RNA-Seq reads from each sample were aligned to the *Glycine max* genome of Williams 82 (Glycine_max_v2.0) [58] via the HISAT2 v2.0.4 with the default options [59]. The transcripts of each sample were reconstructed using String Tie v1.0.4 with options -f 0.3 -j 3 -c 5 -g 100 -s 10000 -p 8 [60]; then, the reconstructed transcripts were compared with reference annotation information using Cuffcompare (one of the tools in Cufflinks v2.2.1) with option -p 12 [61] to identify novel transcripts. The coding potential of the novel transcripts was predicted with CPC v0.9-r2 with the default options [62]. Of the novel coding transcripts, except for the new splicing subtypes of the known genes, the other transcripts belonged to novel genes. After aligning to the reference genome, GATK was used for the SNP calling [63]. Then, the gene expression was quantified via RSEM v1.2.12 with the default options [64]. The significant DEGs were identified by the criteria of at least a 2-fold change, and the *P*-value of the false discovery rate (FDR) correction had to be less than 0.05 between the treated and control groups as determined via DEseq2 [65].

### Gene ontology (GO) and Kyoto encyclopedia of genes and genomes (KEGG) pathway enrichment analysis

All DEGs were annotated with GO categories, including cellular component (CC), biological process (BP) and molecular function (MF) in the database (http://www.geneontology.org/page/go-citation-policy). The calculated enrichment of the GO terms was compared with the genomic background by the hypergeometric distribution, and the *P*-value of the FDR correction had to be less than 0.01.

Similar to the GO analysis, all DEGs were annotated in the KEGG database (http://www.genome.jp/kegg), which is an important public database of pathways that integrates genomics, biochemistry and system functional omics. Using the KEGG pathway as a unit, we compared pathways that were significantly associated with DEGs with the genomic background by computing a *P*-value using the hypergeometric distribution and FDR for multiple testing (*P* ≤ 0.01).

### Analysis of promoter motifs

To analyze promoter motifs, the promoter sequences, which were defined as the 2000 bp region upstream of the predicted transcription start site (TSS), from *Glycine soja* were retrieved (https://www.ncbi.nlm.nih.gov/genome/?term=glycine±soja) [66]. The TF binding sites in the promoters of the chosen genes were predicted via the TF database PlantTFDB version 4.0 (http://planttfdb.cbi.pku.edu.cn/) [67].

### Phylogenetic analyses

We conducted phylogenetic analyses using the coding sequences of regulated TFs. Protein sequences were initially aligned by MUSCLE in MEGA version 7.0 [68]. The amino acid alignments were then used to guide the alignments of the nucleotide coding sequences. Phylogenetic trees were constructed based on the bootstrap neighbor-joining (NJ) method with the default options by MEGA 7.0. The stability of internal nodes was assessed using bootstrap analysis with 1000 replicates.

### Soybean plant transformation

The full-length *GmPT1* cDNA was amplified by PCR based on the sequence information in the NCBI database in accordance with the manufacturer’s protocol for the polymerase used (Phanta Super-Fidelity DNA Polymerase, Vazyme, China) from accession Kefeng No. 1. The *GmPT1* sequence was cloned into the plant binary vector D60005 under the control of the prCaMV 35S promoter with glyphosate resistance (Additional file 20: Figure S7a). The resulting vector containing the target gene and glyphosate resistance was introduced into the *Agrobacterium tumefaciens* strain EHA105 by electroporation, which was then transformed into accession Jack via the cotyledon-node method [69]. Shoots germinated from the cotyledon and eventually regenerated a complete plant. The transgenic plants were validated by PCR amplification (Additional file 20: Figure S7b), and the expression levels of the target and marker genes were analyzed by qRT-PCR. The gene-specific primers were designed via the primer-BLAST website of NCBI (https://www.ncbi.nlm.nih.gov/tools/primer-blast/) and Primer 5.0 software (Additional file 15: Table S13).

## Additional files

Additional file 1: **Table S1.** Number of aligned genes for each sample. (XLSX 10 kb)

Additional file 2: **Table S2.** Non-redundant DEGs. (XLSX 428 kb)

Additional file 3: **Table S3.** DEGs in the susceptible accession W11 at 1 d. (XLSX 190 kb)

Additional file 4: **Table S4.** DEGs in the susceptible accession W11 at 2 d. (XLSX 89 kb)

Additional file 5: **Table S5.** DEGs in the resistant accession W99 at 1 d. (XLSX 210 kb)

Additional file 6: **Table S6.** DEGs in the resistant accession W99 at 3 d. (XLSX 341 kb)

Additional file 7: **Table S7.** GO analysis of DEGs in four comparisons and a common group. (XLSX 13 kb)

Additional file 8: **Table S8.** One hundred forty-six common DEGs in four comparisons. (XLSX 32 kb)

Additional file 9: **Table S9.** GO and KEGG analyses of unique DEGs. (XLSX 13 kb)

Additional file 10: **Table S10.** List of significantly differentially expressed TFs. (XLSX 85 kb)

Additional file 11: **Figure S1.** Phylogenetic analysis of differentially expressed TFs. (a) ERF family; (b) MYB family; (c) WRKY family; (d) bHLH family; and (e) NAC family. Red dots represent the TFs that were up-regulated in one or more comparisons; blue dots represent the TFs that were down-regulated TFs in one or more comparisons; and yellow dots represent the TFs that were oppositely regulated in different comparisons. The gene IDs used in the phylogenetic trees are gene identification number in NCBI database. (DOCX 624 kb)

Additional file 12: **Table S11.** Binding site prediction of representative defense- and growth-related genes. (XLSX 11 kb)

Additional file 13: **Table S12.** Pearson correlation coefficients of gene expression between all samples. (XLSX 14 kb)

Additional file 14: **Figure S2.** Hierarchical clustering of DEGs with three biological replicates, including the treatment and control samples in four groups. (a) Control and treatment samples at 1 d after induction in the resistant line W99; (b) control and treatment samples at 3 d after induction in the resistant line W99; (c) control and treated samples at 1 d after induction in the susceptible line W11; and (d) control and treatment samples at 2 d after induction in the susceptible line W11. The gene expression was quantified via RSEM v1.2.12 with the default options. The values are expressed in FPKM. R1d represents the samples 1 d after induction in W99, R3d represents the samples 3 d after induction in W99, S1 represents the samples 1 d after induction in W11, and S2 represents the samples 2 d after induction in W11. The red and green colors indicate high and low expression levels, respectively. (DOCX 268 kb)

Additional file 15: **Table S13.** Information concerning all primers in the text. (XLSX 15 kb)

Additional file 16: **Figure S3.** Scatter plot of the correlation coefficients between the qRT-PCR and RNA-Seq results of resistant line W99 (a) and susceptible line W11 (b) for early and late induction time points. The x-axis represents log_2_-fold changes of the RNA-Seq data; and the y-axis represents log_2_-fold changes of the qRT-PCR data. (DOCX 58 kb)

Additional file 17: **Figure S4.** Phenotypes of transgenic and control plants. (a) The two plants on the left are Jack control plants, and the two plants on the right are P4-2 plants; (b) the two plants on the left are Jack control plants, and the two plants on the right are P8-2 plants; (c) the CCW larvae before and after feeding on the control and transgenic plants. (DOCX 187 kb)

Additional file 18: **Figure S5.** Representative phenotypes of two select wild soybean and Williams 82 lines. (a) Plant type; (b) size of the trifoliate leaf; (c) characteristics of the seeds; and (d) density of leaf pubescence. (DOCX 449 kb)

Additional file 19: **Figure S6.** Genome mapping statistics of the resistant line (R) and susceptible line (S). (a) Mean of the total clean reads obtained from samples by RNA-Seq; (b) mean mapping ratio aligned to the Williams 82 reference genome; and (c) mean number of total SNPs compared with the number in the reference genome. Statistical significance was detected by a two-tailed t-test. * *P*<0.05; * * * *P*<0.001. (DOCX 95 kb)

Additional file 20: **Figure S7.** Detailed diagram of vector DC60005, including *GmPT1* (a); and identification of transgenic plants by PCR amplification (b). M1: 1000 bp marker; 2: control of H_2_O; 3: control of plasmid; 4: control of nontransgenic plants; 5-9: 5 transgenic plants of line P4; and 10-14: 5 transgenic plants of line P8. (DOCX 180 kb)

## Abbreviations

BP: Biological process
CC: Cellular component
CCW: Common cutworm
CHIP-Seq: chromatin immunoprecipitation coupled with high-throughput sequencing
DEG: Differentially expressed gene
GA: Gibberellic acid
GO: Gene Ontology
JA: Jasmonic acid
KEGG: Kyoto Encyclopedia of Genes and Genomes
MeJA: Methyl jasmonate
MF: Molecular function
PAE: P acquisition efficiency
PUE: P-use efficiency
RIL: Recombinant inbred line
SA: Salicylic acid
SDS: Sequence Detection System
TF: Transcription factor
