# Supplementary material for: RNA-Seq analysis reveals transcript diversity and active genes after common cutworm (Spodoptera litura Fabricius) attack in resistant and susceptible wild soybean lines
Source: BMC Genomics. 2019 Mar 22;20:237. doi: 10.1186/s12864-019-5599-z (PMC6431011; doi:10.1186/s12864-019-5599-z)
Supplement: Supplementary file 11 — Figure S1. Phylogenetic analysis of differentially expressed TFs. (a) ERF family; (b) MYB family; (c) WRKY family; (d) bHLH family; and (e) NAC family. Red dots represent the TFs that were up-regulated in one or more comparisons; blue dots represent the TFs that were down-regulated TFs in one or more comparisons; and yellow dots represent the TFs that were oppositely regulated in different comparisons. The gene IDs used in the phylogenetic trees are gene identification number in NCBI database. (DOCX 624 kb) [file 12864_2019_5599_MOESM11_ESM.docx]

**_
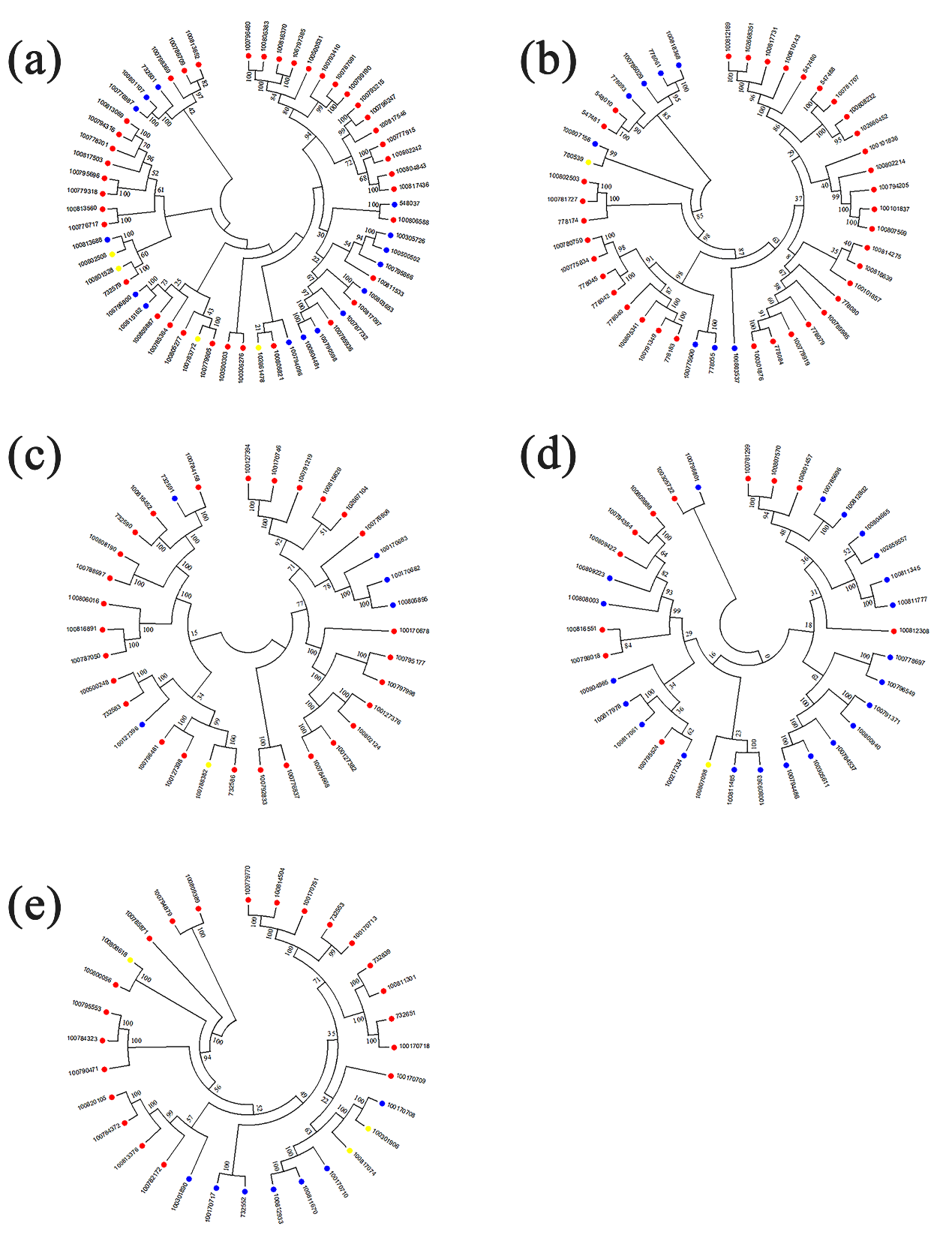
_**

**Additional file 11: Figure S1.** Phylogenetic analysis of differentially expressed TFs. (a) ERF family; (b) MYB family; (c) WRKY family; (d) bHLH family; and (e) NAC family. Red dots represent the TFs that were up-regulated in one or more comparisons; blue dots represent the TFs that were down-regulated TFs in one or more comparisons; and yellow dots represent the TFs that were oppositely regulated in different comparisons. The gene IDs used in the phylogenetic trees are gene identification number in NCBI database.
