# Supplementary material for: RNA-Seq analysis reveals transcript diversity and active genes after common cutworm (Spodoptera litura Fabricius) attack in resistant and susceptible wild soybean lines
Source: BMC Genomics. 2019 Mar 22;20:237. doi: 10.1186/s12864-019-5599-z (PMC6431011; doi:10.1186/s12864-019-5599-z)
Supplement: Supplementary file 14 — Figure S2. Hierarchical clustering of DEGs with three biological replicates, including the treatment and control samples in four groups. (a) Control and treatment samples at 1 d after induction in the resistant line W99; (b) control and treatment samples at 3 d after induction in the resistant line W99; (c) control and treated samples at 1 d after induction in the susceptible line W11; and (d) control and treatment samples at 2 d after induction in the susceptible line W11. The gene expression was quantified via RSEM v1.2.12 with the default options. The values are expressed in FPKM. R1d represents the samples 1 d after induction in W99, R3d represents the samples 3 d after induction in W99, S1 represents the samples 1 d after induction in W11, and S2 represents the samples 2 d after induction in W11. The red and green colors indicate high and low expression levels, respectively. (DOCX 268 kb) [file 12864_2019_5599_MOESM14_ESM.docx]

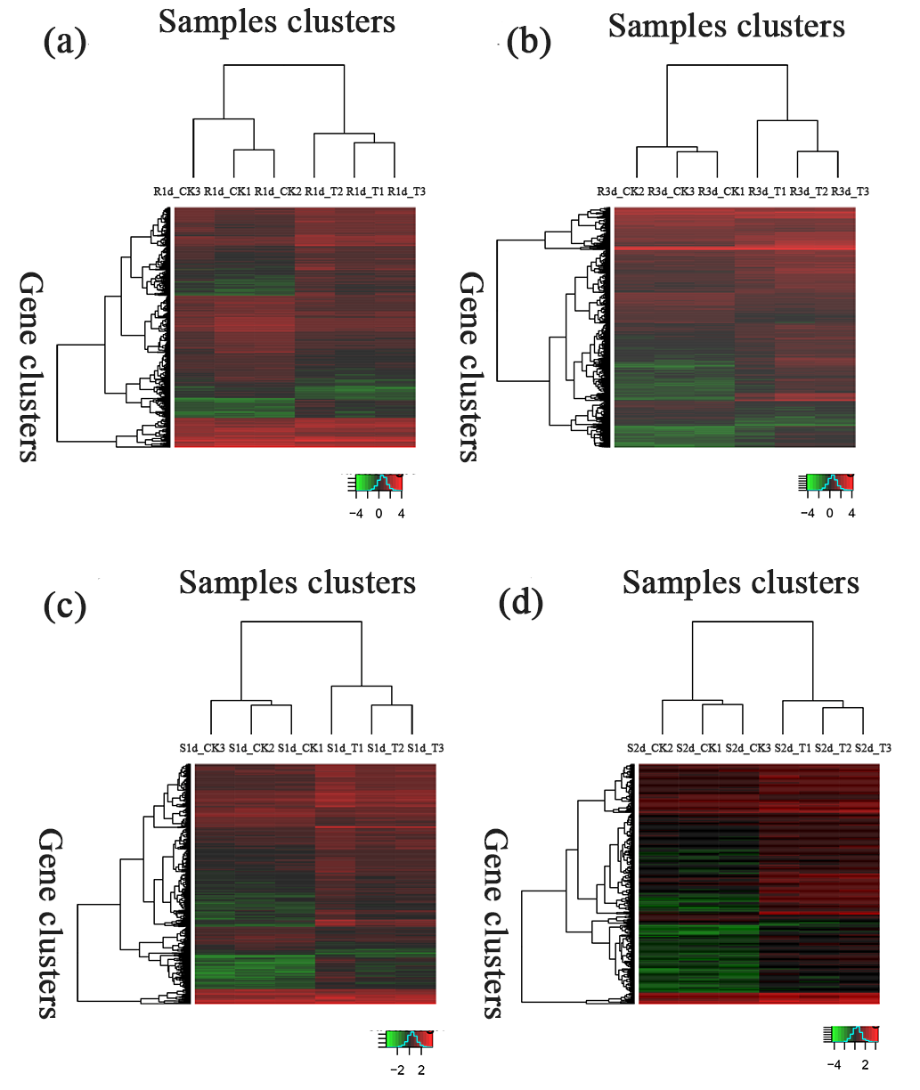


**Additional file 14:** **Figure S2.** Hierarchical clustering of DEGs with three biological replicates, including the treatment and control samples in four groups. (a) Control and treatment samples at 1 d after induction in the resistant line W99; (b) control and treatment samples at 3 d after induction in the resistant line W99; (c) control and treated samples at 1 d after induction in the susceptible line W11; and (d) control and treatment samples at 2 d after induction in the susceptible line W11. The gene expression was quantified via RSEM v1.2.12 with the default options. The values are expressed in FPKM. R1d represents the samples 1 d after induction in W99, R3d represents the samples 3 d after induction in W99, S1 represents the samples 1 d after induction in W11, and S2 represents the samples 2 d after induction in W11. The red and green colors indicate high and low expression levels, respectively.
