# Supplementary material for: RNA-Seq analysis reveals transcript diversity and active genes after common cutworm (Spodoptera litura Fabricius) attack in resistant and susceptible wild soybean lines
Source: BMC Genomics. 2019 Mar 22;20:237. doi: 10.1186/s12864-019-5599-z (PMC6431011; doi:10.1186/s12864-019-5599-z)
Supplement: Supplementary file 16 — Figure S3. Scatter plot of the correlation coefficients between the qRT-PCR and RNA-Seq results of resistant line W99 (a) and susceptible line W11 (b) for early and late induction time points. The x-axis represents log2-fold changes of the RNA-Seq data; and the y-axis represents log2-fold changes of the qRT-PCR data. (DOCX 58 kb) [file 12864_2019_5599_MOESM16_ESM.docx]

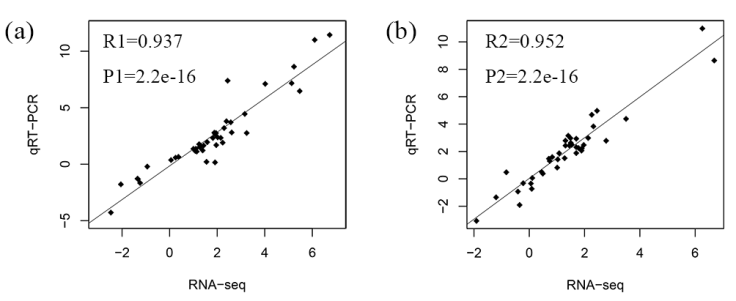


**Additional file 16: Figure S3.** Scatter plot of the correlation coefficients between the qRT-PCR and RNA-Seq results of resistant line W99 (a) and susceptible line W11 (b) for early and late induction time points. The x-axis represents log_2_-fold changes of the RNA-Seq data; and the y-axis represents log_2_-fold changes of the qRT-PCR data.
