# Supplementary material for: RNA-Seq analysis reveals transcript diversity and active genes after common cutworm (Spodoptera litura Fabricius) attack in resistant and susceptible wild soybean lines
Source: BMC Genomics. 2019 Mar 22;20:237. doi: 10.1186/s12864-019-5599-z (PMC6431011; doi:10.1186/s12864-019-5599-z)
Supplement: Supplementary file 17 — Figure S4. Phenotypes of transgenic and control plants. (a) The two plants on the left are Jack control plants, and the two plants on the right are P4-2 plants; (b) the two plants on the left are Jack control plants, and the two plants on the right are P8-2 plants; (c) the CCW larvae before and after feeding on the control and transgenic plants. (DOCX 187 kb) [file 12864_2019_5599_MOESM17_ESM.docx]

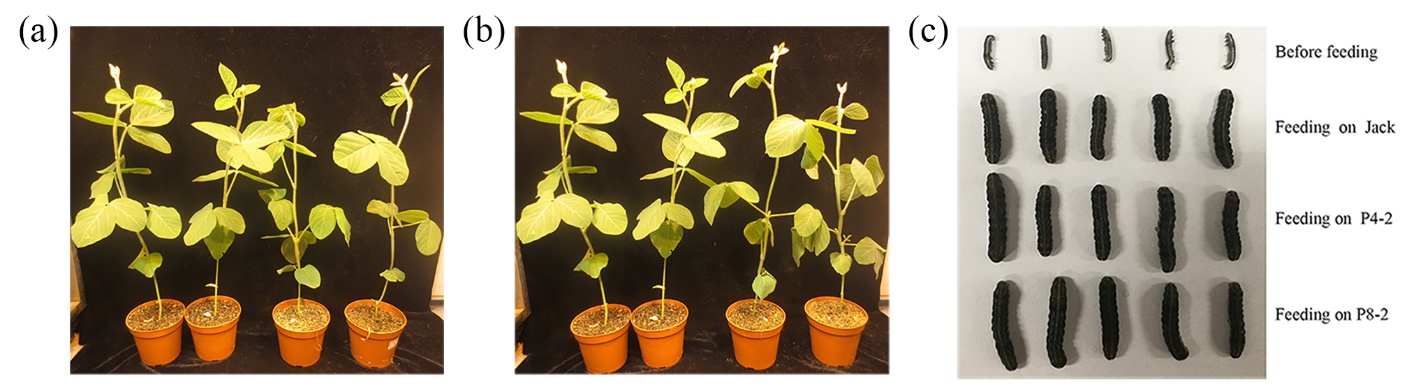


**Additional file 17: Figure S4.** Phenotypes of transgenic and control plants. (a) The two plants on the left are Jack control plants, and the two plants on the right are P4-2 plants; (b) the two plants on the left are Jack control plants, and the two plants on the right are P8-2 plants; (c) the CCW larvae before and after feeding on the control and transgenic plants.
