# Supplementary material for: RNA-Seq analysis reveals transcript diversity and active genes after common cutworm (Spodoptera litura Fabricius) attack in resistant and susceptible wild soybean lines
Source: BMC Genomics. 2019 Mar 22;20:237. doi: 10.1186/s12864-019-5599-z (PMC6431011; doi:10.1186/s12864-019-5599-z)
Supplement: Supplementary file 18 — Figure S5. Representative phenotypes of two select wild soybean and Williams 82 lines. (a) Plant type; (b) size of the trifoliate leaf; (c) characteristics of the seeds; and (d) density of leaf pubescence. (DOCX 449 kb) [file 12864_2019_5599_MOESM18_ESM.docx]

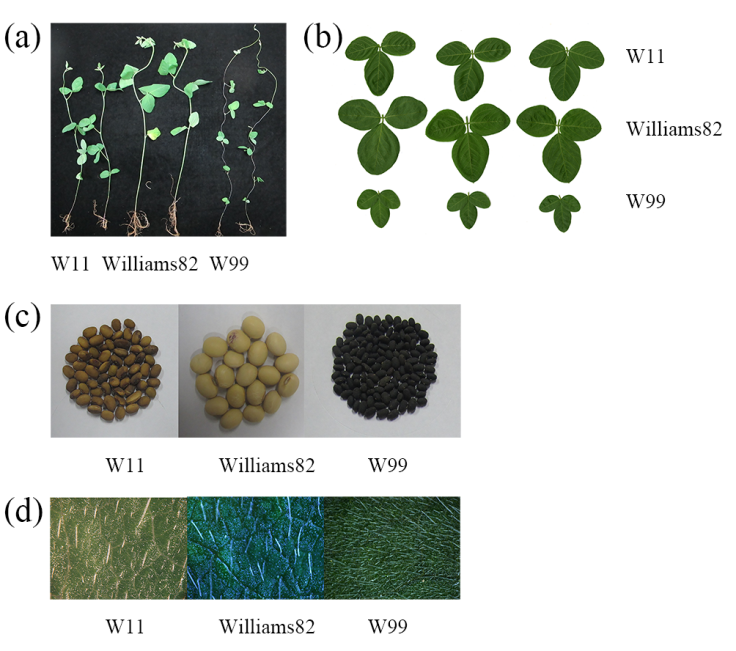


**Additional file 18: Figure S5.** Representative phenotypes of two select wild soybean and Williams 82 lines. (a) Plant type; (b) size of the trifoliate leaf; (c) characteristics of the seeds; and (d) density of leaf pubescence.
