# Supplementary material for: RNA-Seq analysis reveals transcript diversity and active genes after common cutworm (Spodoptera litura Fabricius) attack in resistant and susceptible wild soybean lines
Source: BMC Genomics. 2019 Mar 22;20:237. doi: 10.1186/s12864-019-5599-z (PMC6431011; doi:10.1186/s12864-019-5599-z)
Supplement: Supplementary file 19 — Figure S6. Genome mapping statistics of the resistant line (R) and susceptible line (S). (a) Mean of the total clean reads obtained from samples by RNA-Seq; (b) mean mapping ratio aligned to the Williams 82 reference genome; and (c) mean number of total SNPs compared with the number in the reference genome. Statistical significance was detected by a two-tailed t-test. * P<0.05; * * * P<0.001. (DOCX 95 kb) [file 12864_2019_5599_MOESM19_ESM.docx]

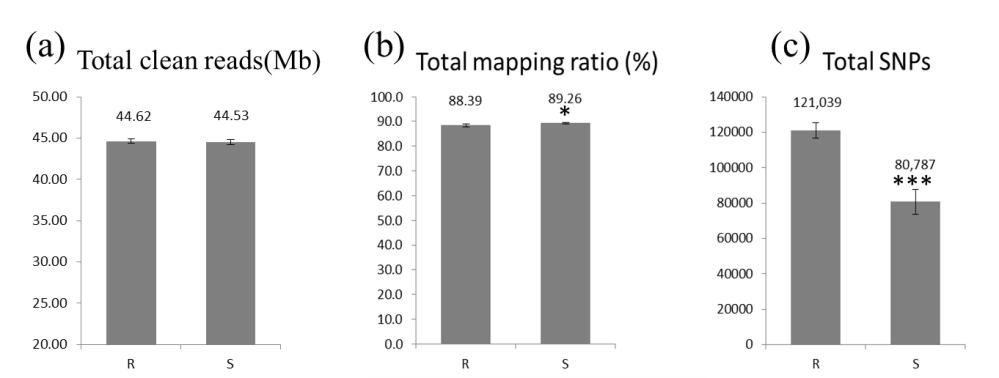


**Additional file 19: Figure S6.** Genome mapping statistics of the resistant line (R) and susceptible line (S). (a) Mean of the total clean reads obtained from samples by RNA-Seq; (b) mean mapping ratio aligned to the Williams 82 reference genome; and (c) mean number of total SNPs compared with the number in the reference genome. Statistical significance was detected by a two-tailed t-test. * *P*<0.05; * * * *P*<0.001.
