# Supplementary material for: RNA-Seq analysis reveals transcript diversity and active genes after common cutworm (Spodoptera litura Fabricius) attack in resistant and susceptible wild soybean lines
Source: BMC Genomics. 2019 Mar 22;20:237. doi: 10.1186/s12864-019-5599-z (PMC6431011; doi:10.1186/s12864-019-5599-z)
Supplement: Supplementary file 20 — Figure S7. Detailed diagram of vector DC60005, including GmPT1 (a); and identification of transgenic plants by PCR amplification (b). M1: 1000 bp marker; 2: control of H2O; 3: control of plasmid; 4: control of nontransgenic plants; 5-9: 5 transgenic plants of line P4; and 10-14: 5 transgenic plants of line P8. (DOCX 180 kb) [file 12864_2019_5599_MOESM20_ESM.docx]

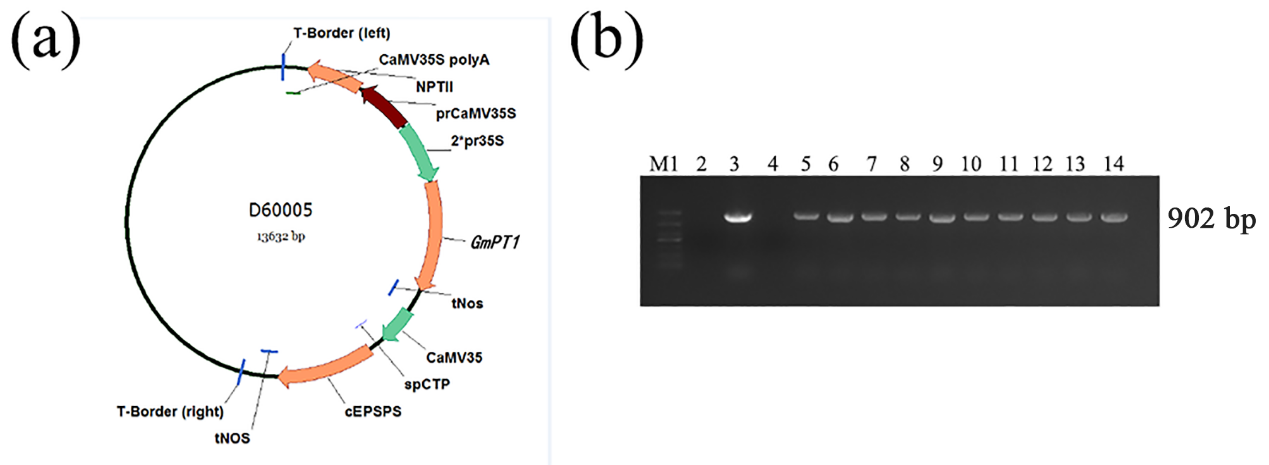


**Additional file 20: Figure S7.** Detailed diagram of vector DC60005, including *GmPT1* (a); and identification of transgenic plants by PCR amplification (b). M1: 1000 bp marker; 2: control of H_2_O; 3: control of plasmid; 4: control of nontransgenic plants; 5-9: 5 transgenic plants of line P4; and 10-14: 5 transgenic plants of line P8.
